# A Novel Cost Based Model for Energy Consumption in Cloud Computing

**DOI:** 10.1155/2015/724524

**Published:** 2015-01-15

**Authors:** A. Horri, Gh. Dastghaibyfard

**Affiliations:** Department of Computer Science & Engineering, College of Electrical and Computer Engineering, Shiraz University, Molla Sadra Avenue, Shiraz 71348 51154, Iran

## Abstract

Cloud data centers consume enormous amounts of electrical energy. To support green cloud computing, providers also need to minimize cloud infrastructure energy consumption while conducting the QoS. In this study, for cloud environments an energy consumption model is proposed for time-shared policy in virtualization layer. The cost and energy usage of time-shared policy were modeled in the CloudSim simulator based upon the results obtained from the real system and then proposed model was evaluated by different scenarios. In the proposed model, the cache interference costs were considered. These costs were based upon the size of data. The proposed model was implemented in the CloudSim simulator and the related simulation results indicate that the energy consumption may be considerable and that it can vary with different parameters such as the quantum parameter, data size, and the number of VMs on a host. Measured results validate the model and demonstrate that there is a tradeoff between energy consumption and QoS in the cloud environment. Also, measured results validate the model and demonstrate that there is a tradeoff between energy consumption and QoS in the cloud environment.

## 1. Introduction

Cloud computing provides IT services to users via the Internet. Cloud computing enables the hosting of pervasive applications. It is based upon a pay-as-you-go model; therefore, it appeals to business owners. For instance, it allows for the gradual progress of an enterprise from small-scale to large-scale resources.

One of the differences between cloud computing infrastructure and other computing infrastructures is the deployment of virtualization technology. Hence, the cloud has a virtualization layer, while the other computing systems (i.e., Grid) do not have such a layer [1]. Traditional application provisioning models assign individual application elements to computing nodes. A good illustration of a cloud is a host that has a single processing core. There is a requirement of concurrently instantiating two VMs on that host. Even though in practice VMs are context isolated, the VMs need to share the processing cores. Thus, the amount of hardware resources available to each VM is constrained by the total hardware resources available within the host. CloudSim simulator [2] supports VM provisioning at two levels: host level and VM level. At host level, it is possible to specify how much of the total processing power of each core will be assigned to each VM. At VM level, the VM assigns a fixed amount of available processing power to the individual application services (task units).

One of the cloud benefits is the possibility to dynamically adapt (scale-up or scale-down) the amount of (provisioned) resources to applications in order to attend the variations in demand, which are predictable [3]. Elastic (automatic scaling) applications, such as web hosting, content delivery, and social networks, can use this cloud ability, which is susceptible to elasticity.

Green cloud computing is developed not only to achieve efficient processing and utilization of computing resources, but also to minimize energy consumption [4]. This is essential for sustainable growth of cloud computing [5]. Otherwise, data centers will cause a considerable increase in energy consumption [6]. To achieve Green cloud computing, data center resources need to be managed; that is, cloud resources need to be allocated not only to satisfy QoS requirements (specified by users via service-level agreements (SLA)), but also to reduce energy consumption [7].

Although modern virtualization technologies can ensure the performance isolation between VMs, which share the same host, due to the aggressive consolidation and the variability of the workload, some VMs may not obtain the required amount of resources when requested. This leads to decreasing performance in terms of increased response times, timeouts, or failures in the worst case. Therefore, cloud providers have to deal with the power-performance tradeoff (i.e., minimizing energy consumption while meeting the QoS requirements). In this work, the cost and energy usage of time-shared policy was measured and then the cost was modeled in the CloudSim simulator. Based on the proposed model, various scenarios were simulated and the results indicated that the energy consumption is considerable. Hence, cloud providers have a tradeoff between the response time and time-shared policy cost.

One of the important requirements to be provided by cloud computing environments is reliable QoS [8]. It is defined in terms of the service-level agreements (SLA), which describe such characteristics as the throughput, response time, or latency delivered by the deployed system [9]. Response time is an amount of time, obtained from the interval between the request submission and the first-response production. In interactive systems such as web services and real-time applications, response time is the major QoS factor [10]. Response time is a function of load intensity, which can be measured in terms of arrival rates (such as requests per second) or the number of concurrent requests. In the situation where the response time is more important than the other QoS factors, the system must apply time-shared policy. The rest of the paper is organized as follows: first we present related work and our main motivations for this work in Section 2.  Section 3 introduces the measuring context switch cost. Experimental results are then detailed in Section 4, and finally Section 5 concludes the paper.

## 2. Literature Review

### 2.1. Time-Shared and Space-Shared Policy

**Figure 1:**
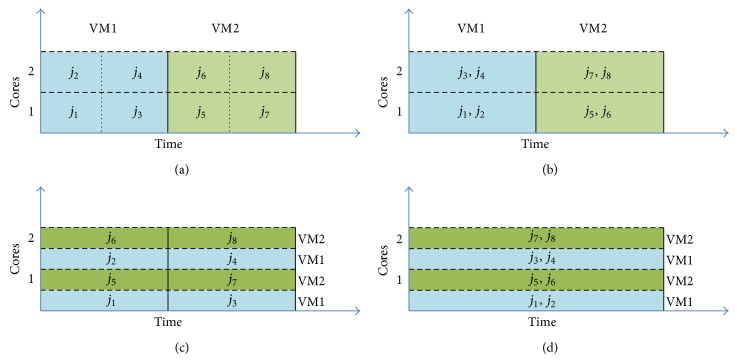
Time-shared and space-shared policy for VMs and jobs. (a) Space-share for VMs and jobs, (b) space-share for VMs and time-share for jobs, (c) time-share for VMs and space-share for jobs, and (d) time-share for VMs and jobs.

Some virtualization tools like XEN support time-shared policy [11] and space-shared policy. CloudSim can apply the time-shared or space-shared provisioning policies at VM or host level. In time-shared policy resources will not be released until is finished and no context switch is used. The difference between these policies has been shown in Figure 1, which demonstrates a simple VM provisioning scenario. In this figure, a host with two CPU cores receives a request for the hosting of two VMs, such that each one requires two cores and plans to host four jobs. More specifically, jobs *j*_1_, *j*_2_, *j*_3_, and *j*_4_ are to be hosted in VM1, while *j*_5_, *j*_6_, *j*_7_, and *j*_8_ are to be hosted in VM2.

In Figure 1(a) the space-shared policy is applied for both VMs and jobs. In this mode, only one VM can run at a given instance of time since each VM requires two cores. Therefore, VM2 can only be assigned to the core after VM1 has finished the execution of task units. The same happens for the provisioning jobs within the VM1: since each job unit demands only one core, both of them can run simultaneously. During this period, the remaining jobs (3 and 4) wait in the execution queue.

Figure 1(b) presents a provisioning scenario, where space-shared policy is applied for allocating VMs to hosts and a time-shared policy is applied to allocate job units to the processing core within a VM. As a result, during a VM lifetime, all the assigned jobs are dynamically context switched during their life cycle.

In Figure 1(c), a time-shared policy forms the basis for allocating VMs to hosts, while space-shared policy is used for jobs in this scenario; each VM receives a time slice on each processing core and then distributes the slices to job units on a space-shared basis. As cores are shared, the amount of processing power available to a VM is variable.

Finally, Figure 1(d) presents a time-shared policy for both VMs and job units. Hence, the processing power is shared by VMs, and within each VM jobs are executed in time-shared policy.

As expected, in the space-shared mode, the arrival of new tasks does not have any effect on the tasks under execution, and every new task is queued. However, in the time-shared mode, the execution time of each task varies with an increase in the number of submitted tasks. Allocating tasks to VMs by time-shared policy has a significant effect on execution times, as the processor is remarkably context switched among the active tasks. Hence, when the time-shared policy is used, cost and energy consumption of context switches must be taken into account.

### 2.2. Green Cloud

There are many research papers on energy saving in the cloud computing [12–14]. Some proposed approaches [15–17] are based on VMs consolidation and minimizing the number of nodes by switching off idle nodes or switching them to low power states. The main goal of VM consolidation is to increase the utilization of hosts and reduce energy consumption by reallocating VMs using live migration. Horri et al. [18] proposed a VMs consolidation approach for cloud environments that adopts a method based on the resource utilization history of virtual machines. The simulation results demonstrated that there is a tradeoff between energy consumption and quality of service in the cloud environments. VMs consolidation technique reduces power consumption and increases resource utilization. However, minimizing power consumption may lead to SLA violation and also lead to increase in system overhead [19]. Also the above approaches did not consider the overhead when multiple VMs are on a host. As it has been stated in [20], the amount of resources consumed by a single VM on a host is different with multiple VMs. Since the hypervisor and host OS require additional resources (e.g., CPU or I/O bandwidth during resource scheduling) and VMs instances may interfere with each other, they consume a higher amount of a shared resource such as cache during context switching. A hypervisor or virtual machine monitor (VMM) is software or firmware that creates, runs, and manages virtual machines.

In a multitask system, the context switch refers to the switching of CPU from one process to another. Context switch allows processes to execute concurrently. Measuring the cost of the context switch is a challenging problem. The cost of time-shared policy has not been modeled in the cloud literature. Hence, the context switch cost should be measured in real systems and then this measurement is used to model the cost of the time-shared policy in the CloudSim.

The context causes unavoidable system overhead. The cost of the context switch may come from several aspects. Direct costs consist of saving and restoring processor registers, executing the OS kernel code (scheduler), reloading the TLB, and flushing the processor pipeline [21]. These costs are directly associated with almost every context switch in a multitasking system. In addition, context switches lead to the sharing of the cache among multiple processes [22]. This may result in performance degradation. This cost varies with different architectures and different workloads which have different memory access patterns [23]. This cost is referred to as the cache interference cost.

In [24, 25], the direct cost of the context switch was measured using a benchmark with two processes communicating with each other via two pipes. In our work, in order to measure the cost of the context switch, the pipe communication was also used to implement the frequent context switches between two processes. The direct time cost per context switch (c1) was measured using Ousterhout's method [24], where processes are not involved in data access. In [26], based on Ousterhout's method, the cost of the direct and indirect context switch was measured. The authors used the pipe for their measurements.

### 2.3. Power Model

**Figure 2:**
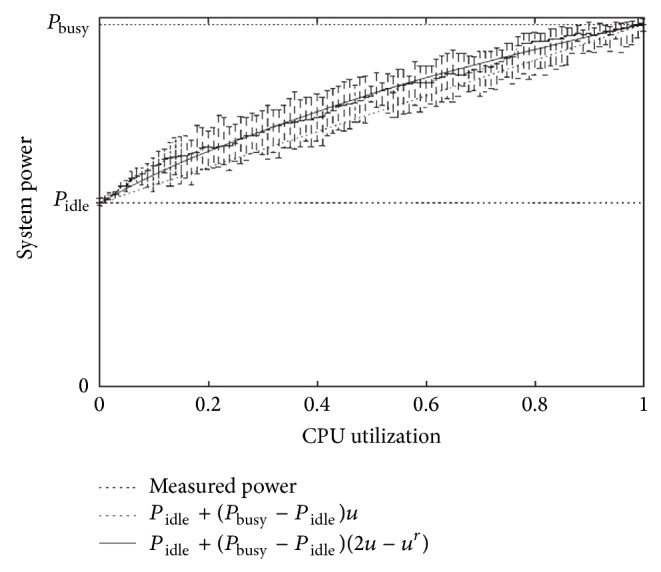
Power consumption and CPU utilization relationship [27].

Fan et al. [27] have found a strong linear relationship between the system CPU utilization and total power consumption of the system. As Figure 2 shows, the proposed model of them shows that the power consumption of a system grows linearly with the growth of the CPU utilization.

Based upon [28, 29], applying DVFS on CPU results in an approximately linear power-to-frequency relationship on average an idle server consumes approximately up to 74% of the power consumed by the server running at 100% CPU utilization.  Table 1 shows the percentage of power consumption in idle and full CPU utilization for some servers and it is based on spec data [30].

In [31, 32], the authors have proposed the following model for power and energy of CPU in the cloud:

(1) $$\begin{array}{c} P\left(u\right)=k\times P_{\max}+\left(1-k\right)\times P_{\max}\times u, \end{array}$$

where *P*_max⁡_ is the maximum power consumed when the server is fully utilized, *k* is the fraction of power consumed by the idle server, and *u* is the CPU utilization. The utilization of CPU may change over time due to workload. Thus, the CPU utilization is a function of time and is represented by *u*(*t*). Therefore, the total energy (*E*) consumption by a physical host can be calculated as an integral of the power consumption function over a period of time:

(2) $$\begin{array}{c} E=\overset{\;}{\underset{t}{\int }}P\left(u\left(t\right)\right). \end{array}$$

In this study, power model defined in (1) has been used.

To the best of our knowledge, no research has been carried out on the measurement of the context switch cost, as well as modeling energy consumption of time-shared policy in the CloudSim.

Energy consumption by computing hosts in data centers consists of that of CPU, disk storage, and network interfaces. A strong linear relationship exists between the system CPU utilization and total power consumption of the system [27]. This work has focused on measuring and modeling CPU energy consumption in time-shared policy.

### 2.4. CloudSim Simulator

It is important to measure the cost on a large-scale cloud infrastructure. However, it is hard to manage large-scale experiments on a real infrastructure, especially when it is essential to repeat the experiments with the same conditions (e.g., comparing different scenarios). So, simulations have been selected as a way to evaluate the proposed model.

CloudSim is a platform in which cloud strategies can be tested in a controlled and reproducible manner [2]. Therefore, simulation frameworks such as CloudSim are important, as they allow for the evaluation of the performance of resource provisioning and application scheduling techniques under different infrastructure configurations.

## 3. Measuring Context Switch Cost

In this section, the proposed method is introduced. The method is based on the data measured at a number of observations on the real system. In the first subsection, the real system model is described and in the second subsection the model that is used for simulation is depicted.

### 3.1. Measuring Context Switch Cost in Real System

According to Ousterhout's method [24], two processes repeatedly send a single-byte message to each other via two pipes. In each round-trip communication, two context switches as well as one read and one write system call in each process will occur. In this study the time cost of 1,000 round-trip communications (*t*_1_) was measured, and the time cost of 1,000 simulated round-trip communications (*t*_2_), which includes no context switch cost, was measured as well. The direct time cost per context switch was calculated as *c*_1_ = *t*_1_/2000 − *t*_2_/1000.

A dual-core processor was utilized in the experiment to avoid the potential interference from OS interrupt handling or from other processes. The communicating processes in the experiment were assigned to the one core. The real-time scheduling policy was also set by setting SCHED_FIFO and giving them the maximum priority.

The machine in this experiment had a CPU with dual 2.0 GHz Intel Pentium cores with 2 MB cache. The average direct context switch cost in our system was 3.9 microseconds.

To measure the effect of data access, after each process had been run, it accessed an array of long integers (long long int) before writing a message to the other process and then was blocked on the next read operation. The total data accessed by each process (array size) changed during the different test runs. The experiment results fall into three categories. In the first category, the cost is relatively constant, with context switch times ranging from 4 *μ*s to 8.2 *μ*s. This is because the entire datasets of the two communicating processes can fit into the cache, and the context switch did not cause any considerable cache interference. In the second category, the dataset of one process fits in the cache, but the combined datasets of the two communicating processes do not; the cost of the context switch increases dramatically, from 35 *μ*s to 190 *μ*s, with the increase of the array size. For each context switch, it is essential that the newly scheduled process refill the L2 cache with its own data. With regard to the third category, both datasets cannot fit into cache. The cost of the context switch is still high, compared to the first category, showing the presence of cache interference, whereas the cost does not increase monotonously in comparison with the second category.

In this study, based upon the obtained results, the cost and energy usage of time-shared policy was modeled in the CloudSim and then this model was evaluated by various scenarios.

### 3.2. Modeling Context Switch in Simulator

In order to model the context switch cost, the CloudSim simulator has been extended. Based on the result of the real system modeling, the context switch time was calculated. To model the context switch in the simulator, the number of instructions per context switch (IPC) was needed. To calculate IPC, we used IPS (instructions per second) of the processor, which depends on CPU architecture and is available in the processor specifications table. One of the important parameters influencing the cost of the context switch is the context switch frequency. To model it, a quantum parameter was defined, which showed the number of instructions that executing between two context switches in MIPS (million instructions).

Hence, indirect cost of context switches is

(3) $$\begin{array}{c} \text{ICost}\left(J\right)=\sum \left(\left(\frac{I\left(j\right)}{\left(q\times \text{IPS}\right)}\right)\times t\right)\;\forall j\in J, \end{array}$$

where *J* is the set of jobs to be executed in the time-shared policy, IPS is the processor instructions per second, *t* is the cost of a context switch in MI (million instructions), *I*(*j*) is the total instructions for job *j* in MI, and *q* is the quantum parameter in the second.

And the total cost of context switches is

(4) TCost=ICost,if  D<C,∑Ijq×IPS×T(D),if  D>C, di<C,lllllllllllllllll∀j∈J,∑Ijq×IPS×tmax⁡,if  di>C ∀j∈J,TD=D×tmax⁡−tmin⁡C,D=∑di  di=data  size  of  job  i ∀i∈J,

where *C* is the cache size, *D* is the sum of the data size of all processes in KB, *T*(*D*) is a linear MI function based on *D* when *D* is larger than the cache size but the data size of each process is smaller than the cache size, and *t*_min⁡_ is the minimum and *t*_max⁡_ is the maximum context switch cost obtained in this situation per MI. Tcost is the total cost of time-shared policy in MI.

In the Tcost function, the first row indicates that all data can fit in the cache. Hence, the cache interference does not exist. The second row is related to a situation where all data cannot fit in the cache, but the individual data of each process can fit in the cache. This is modeled as a linear function. The last row shows that the data of each process cannot fit in the cache. In this case, the cost of the context switch is at the maximum value.

Equation (5) is proposed to measure extra energy consumption by the time-shared policy. This equation uses the linear model described in (1) and (2). Consider

(5) $$\begin{array}{c} E_{\text{CS}}=\overset{t=(\text{TCost}/\text{vmmips})\times (1/u)}{\underset{t=0}{\int }}P\left(u\right)dt, \end{array}$$

where *P*(*u*) is the power consumed by the host, vmmips indicates the processor speed of the VM in MIPS (million instructions per second), TCost is the cost of the time-shared policy in MI, and *u* is the CPU utilization. Hence, TCost/vmmips × *u* is the extra time for the time-shared policy. *E*_CS_ will be the extra energy usage should the time-shared policy be applied.

Every job in the cloud must be executed on a VM using time-shared policy. Hence, for each job the cost and energy usage is calculated by the VM parameters based on the abovementioned methods.

Equation (6) is also proposed to measure total energy consumed by a host, where *E* is the energy consumed when space-shared policy is applied:

(6) $$\begin{array}{c} E_{\text{total}}=E_{\text{CS}}+E. \end{array}$$

## 4. Experimental Result

In this section, we will discuss analysis of the model described in the previous section. As the targeted system is a cloud computing environment, it is essential to evaluate it on a large-scale infrastructure. Hence, a data center with 500 heterogeneous physical hosts was simulated. Each host was modeled to have a dual core CPU; the performance of each core thereof is equivalent to 1000 million instructions per second (MIPS), 4 GB of RAM, 2 MB of cache memory, and 1 TB of storage. The power consumption by the hosts was defined according to the model described in the previous section. Based on this model, a host consumes power from 210 W with 0% CPU utilization up to 300 W with 100% CPU utilization. Each VM requires one CPU core with 250 MIPS, 128 MB of RAM, and 1 GB of storage. The users submit requests for the provisioning of 1000–8000 heterogeneous VMs. To model the CPU utilization, each VM runs a web application that uses a uniformly distributed random variable workload and requires 10,000–200,000 MIPS. The results are based on the mean value of running each experiment 5 times.

Tables 2 and 3 show the cloud configurations for theses experiment.

**Figure 3:**
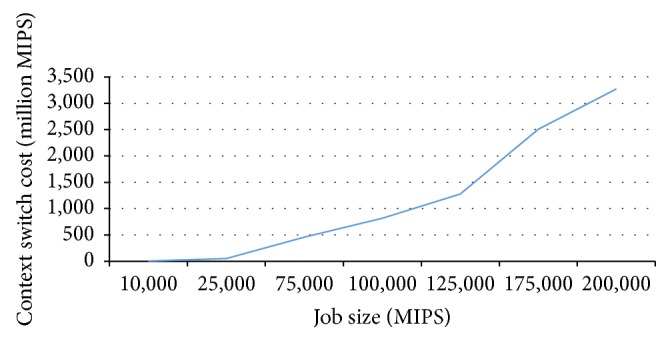
Indirect context switch cost and the job size.

**Figure 4:**
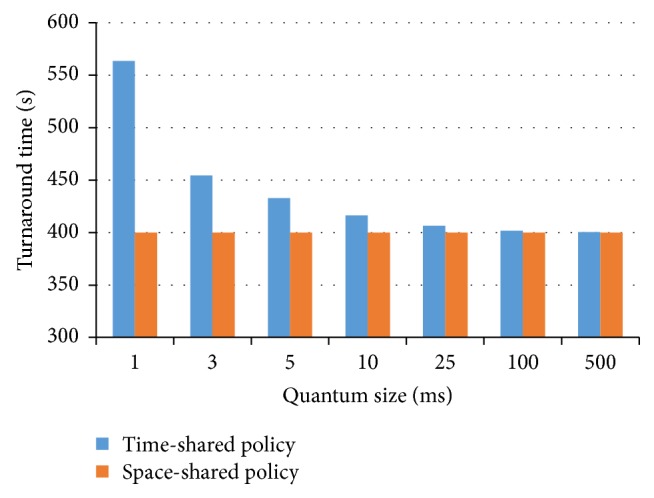
Indirect context switch cost and the size of quantum parameter.

**Figure 5:**
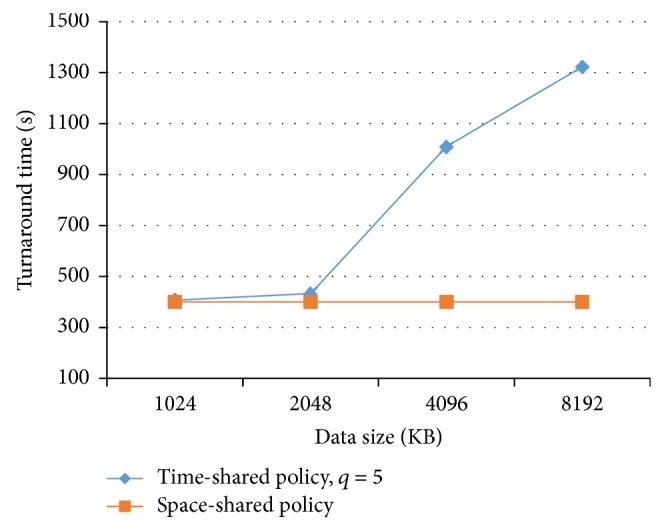
Turnaround time and data size.

In Figures 3, 4, and 5 the dataset size of applications is zero. Hence, these charts demonstrate the indirect cost.

Figure 3 demonstrates the cost of time-shared policy as the size of the jobs is increased. In this figure, axis *X* represents job size and axis *Y* represents the extra cost caused by context switch overhead, given in MIPS. As can be seen, if the job size increases, the cost of time-shared policy will also increase. This is due to the fact that the larger jobs cause more context switches than smaller jobs. In the cloud environment, the job size is sufficiently large. In this case, the quantum size is 5 msec.

Next experiment was planned to evaluate the effect of the quantum parameter on the turnaround time for time-shared policy. Turnaround time is the amount of time between the submission of a job and its completion. From the point of view of a particular job, the important criterion is how long it takes to execute that job. In a real-time or interactive system, such as some application in geospatial sciences, use of turnaround time may not be a good measure [33]. Often, a job can produce some results and output them, and then it can continue to produce new results while previous results are being output to the user. Hence, another criterion is the total time from the submission of a job until the first response is produced (response time).  Figure 4 shows that the turnaround time of jobs in the time-shared policy increases as the quantum parameter decreases. In this experiment, the job length is 100,000 MI and the number of VMs is 4,000. An increase in the quantum parameter contributes to a decrease in the time-shared policy cost. Calculating the optimal amount of quantum parameter is a multicriteria optimization problem.

Increasing the quantum size will decrease the turnaround time and increase average response time (time from submission till the first response is produced), whereas a decrease in the quantum parameter leads to a decline in the response time and a rise in the time-shared policy cost. Hence there exists a tradeoff between the cost of the context switch and the response time. In the cloud environment, both the cost and the response time are important. Therefore, it is required that the optimal values be selected for this parameter. If the quantum parameter is sufficiently large, then the time-shared policy will become the space-shared policy. In this situation, in a time slice a job can obtain the total time necessary for execution.

### 4.1. Effect of Data Access

In this section, the data access impact is shown. As can be seen, the data access cost of the time-shared policy may be high.

Figure 5 shows the data size increment effect in the turnaround time when the quantum parameter is 5 msec. The time-shared policy curve shape is consistent with Section 3 results.

The time-shared policy curve has three regions. In the first region the curve is relatively flat. This is because the entire datasets of processes can fit into the cache, and the context switch does not cause any considerable cache interference. In the second region the dataset of one process fits in the cache, but the combined dataset of all processes does not; the cost of the context switch increases dramatically with the increment of the array size. For each context switch, it is essential that the newly scheduled process refill the L2 cache with its own data. In the third region any datasets cannot fit into cache. The cost of the time-shared policy is still high as compared to the first region, showing the presence of cache interference.

**Figure 6:**
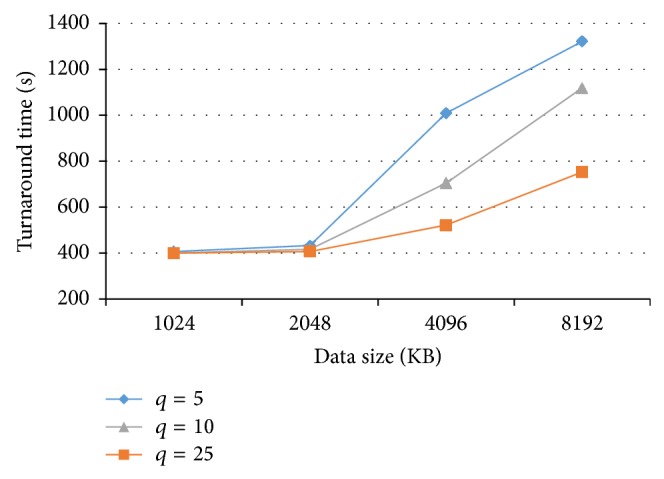
Turnaround time for various quantum parameters with increasing data size.

Figure 6 demonstrates the turnaround time for various quantum parameters as the data size increases. As can be observed, the quantum parameter is essential for the time-shared policy cost. The cost will increase dramatically, on condition that the quantum parameter is small and that the data size is higher than that of the cache. Hence, in order to decrease the time-shared policy cost, the quantum parameter must be large enough, leading to an increase in the response time. Therefore, there will be a tradeoff between the cost and the QoS in this situation.

**Figure 7:**
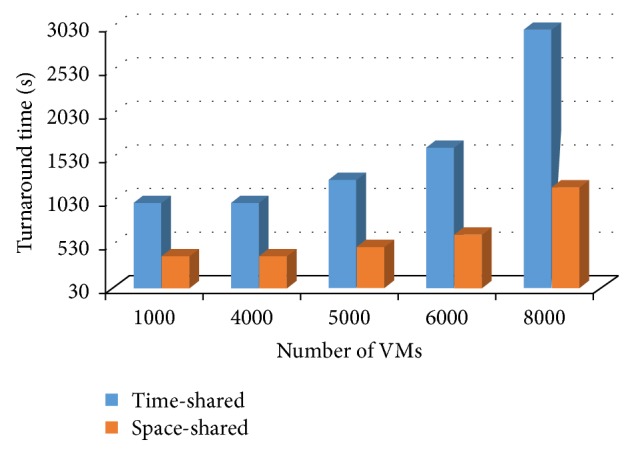
Turnaround time and the amounts of VMs.

Next experiment was planned to evaluate the effect that the host VMs number has on the turnaround time in time-shared policy in comparison with space-shared policy.  Figure 7 shows that the turnaround time of jobs in the time-shared policy increases versus space-shared policy as the amount of VMs increases. This is due to the fact that a larger number of VMs cause more context switches than small number of VMs. In the cloud environment, the number of VMs is sufficiently large. In this experiment, the job length is 100,000 MI and the *q* is 5 msec. So, if cloud providers decide to consolidate VMs to reduce energy consumption, then they have to consider the cost of VMs consolidation.

### 4.2. Energy Consumption

In this section, the time-shared policy impact on the data center energy consumption is shown.

**Figure 8:**
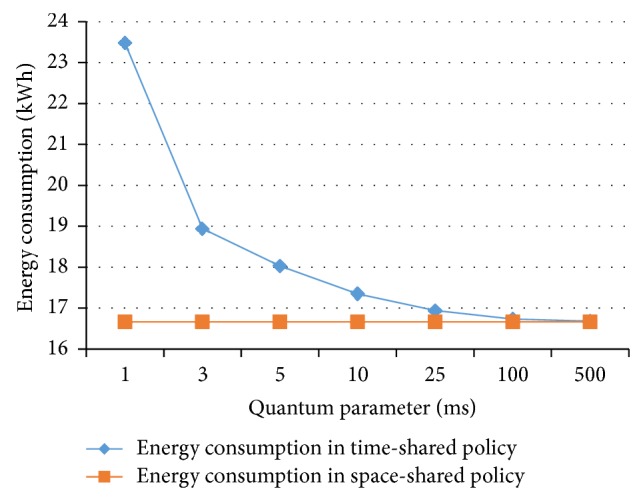
Energy consumption and quantum parameter.

The first experiment was designed to show the effect of quantum parameter on the energy consumption in time-shared policy.  Figure 8 shows that the energy consumption of jobs in the time-shared policy increases as the quantum parameter decreases. In this experiment, the job length is 100,000 MI and number of VMs is 4,000. An increase in the quantum parameter contributes to a decrease in the time-shared policy energy consumption.

**Figure 9:**
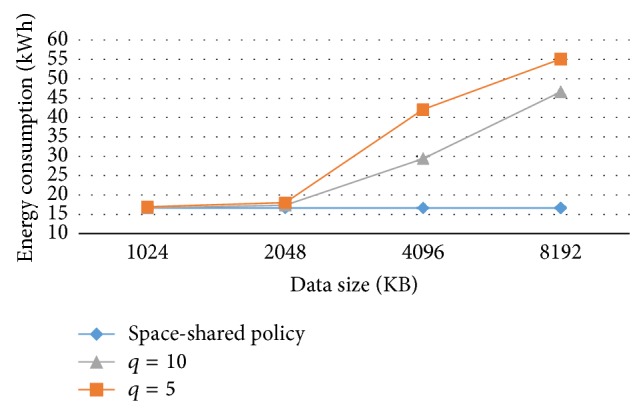
Energy consumption and data size with various quantum parameters.

As can be seen in Figure 9, the data center energy consumption in the time-shared policy may be high. The energy consumption is very high, provided that the data size is larger than the cache size and that the quantum parameter is small.

Like the previous section, these curves consist of three parts as well. In the first part the total datasets of all processes can fit in the cache. In the second part the dataset of all processes cannot fit in the cache, but the dataset of one process can fit in the cache. In the third part no dataset can fit in the cache. Thus, this causes more context switches and more cache misses.

**Figure 10:**
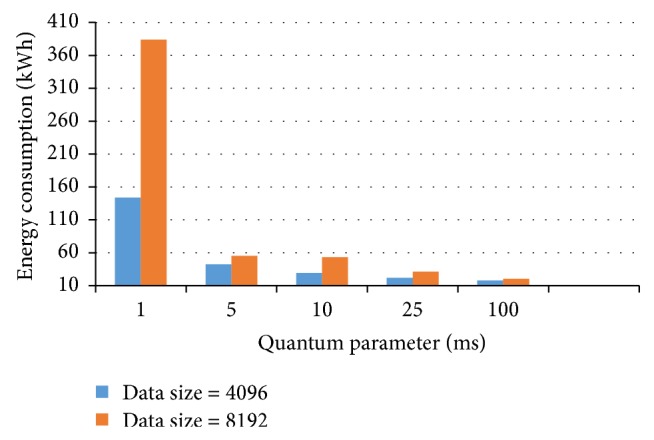
Energy consumption and quantum parameter with two dataset sizes.

Figure 10 demonstrates the effect of the quantum parameter in energy usage. The cost is high, providing that the quantum parameter is small and that the data size does not fit in the cache.

If the quantum parameter equals one, then the cost will be huge due to thrashing. Thrashing means that the CPU will first do a small part of Job 1, then a small part of Job 2, and finally Job *n*; then again, it will do a small part of Job 1, then Job 2, and finally Job *n*; and this scenario is repeatedly carried out. This context switching leads to thrashing, and the operating system spends more time on switching jobs rather than actually performing jobs and the cache miss rate increases substantially. To avoid this situation, the quantum parameter must be tuned accurately because the quantum parameter increase can have an impact on the response time.

**Figure 11:**
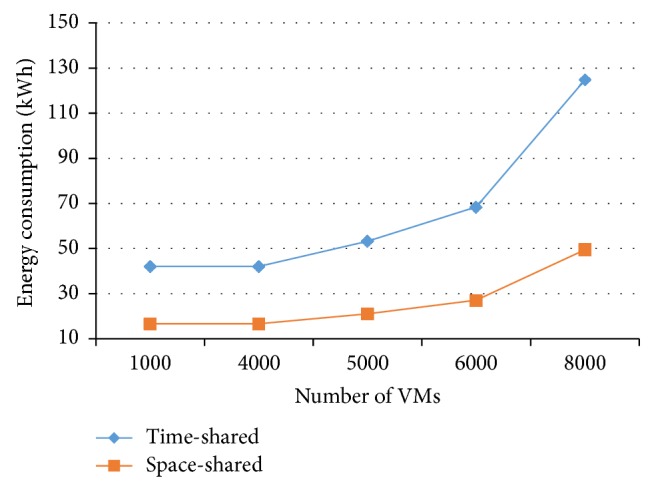
Energy consumption and amounts of VMs.

Figure 11 demonstrates the effect that the host VMs number has on the energy consumption in time-shared policy in comparison with space-shared policy. This figure shows that the energy consumption in the time-shared policy increases versus space-shared policy as the amount of VMs increases. In this experiment, the job length is 100,000 MI and the *q* is 5 msec. Hence, if cloud providers decide to consolidate VMs to reduce energy consumption, then they have to consider this extra energy consumption.

## 5. Conclusion

Cloud providers have to make a tradeoff between energy consumption and SLAV. In interactive systems such as web services and real-time applications, the response-time is the main QoS factor. To guarantee the response time, the time-shared policy must be applied to allocate job units to the processing core within a VM. As a result, during the VM lifetime, all the assigned jobs are dynamically context switched during their life cycle. This switching has considerable costs such as saving and restoring the processor registers, executing the OS kernel code (scheduler), reloading the TLB, and causing the cache interference. This paper describes the approach to and the result of the measurements and model on the total cost and energy consumption of the time-shared policy in cloud environments. In this study, based upon the results obtained from the real system, the cost and energy usage of time-shared policy was modeled in the CloudSim simulator and then this model was evaluated by various scenarios. Results indicated the following.

- The energy consumption may be so high and it can vary with different parameters such as the following.
  - The quantum parameter: if the quantum parameter is small, then the cost is high. On the other hand, if the quantum parameter is large, then time-shared policy will become the space-shared policy.
  - Data size: if the data size is larger than the cache size, then the energy consumption is very high.
  - Number of VMs: the energy consumption increases as the number of VMs increases.
- Results indicate that the extra energy can be as much as 150 percent of space-shared policy in the worst case. So, cloud providers have to consider this cost, when they decide to consolidate VMs (for energy efficiency purpose).

In this study, the data access pattern was sequential. This research can be extended by investigating the cost of other data access patterns. The cost may depend on the types of application to be run on the host. Hence, analyzing the energy consumption based upon the workload type can be interesting. Also, we are looking at extending our model to use it in VMs consolidation algorithms. Also, we plan to use the proposed models in a real cloud infrastructure such as OpenStack [34].

## Figures and Tables

**Table 1:** Percentage of power consumption in idle and full CPU utilization.

	Computer model	CPU model	Power at idle (0%)	Power at full (100%)	Idle/Full
1	Super micro 6025B-TR+	L5335	223	300	74%
2	HP ProLiant DL160 G5	L5420	148	233	66%
3	Acer Altos R720	L5430	135	206	65%
4	Super micro 6025B-TR+	E5345	219	334	65%
5	Acer AT150 F1	X5670	65	224	30%

**Table 2:** Host Parameters.

	Host parameter in data center	Value
1	Number of hosts	500
2	Type of CPU	Dual core
3	Each core performance	1000 MIPS
4	RAM	4 GB
5	Cache memory	2 MB
6	Storage	1 TB
7	Power consumption by each host	210–300 W

**Table 3:** VM Parameters.

	VM parameter	Value
1	CPU	one CPU core with 250 MIPS
2	RAM	128 MB
3	Storage	1 GB
4	Number of VMs	1000–8000
